# Population-based study of long-term anticoagulation for treatment and secondary prophylaxis of venous thromboembolism in men with prostate cancer in Sweden

**DOI:** 10.1186/s12894-022-00967-z

**Published:** 2022-02-02

**Authors:** Yanina Balabanova, Bahman Farahmand, Pär Stattin, Hans Garmo, Gunnar Brobert

**Affiliations:** 1 Integrated Evidence Generation, Bayer AG, 13353 Berlin, Germany; 2 Integrated Evidence Generation, Bayer AB, Stockholm, Sweden; 3 Department of Surgical Sciences, Urology, Uppsala University, Uppsala, Sweden

**Keywords:** Anticoagulants, Bleeding, Prostate cancer, Venous thromboembolism

## Abstract

**Background:**

Epidemiological data on anticoagulation for venous thromboembolism (VTE) in prostate cancer are sparse. We aimed to investigate associations between anticoagulation duration and risks of VTE recurrence after treatment cessation and major on-treatment bleeding in men with prostate cancer in Sweden.

**Methods:**

Using nationwide prostate cancer registry and prescribing data, we followed 1413 men with VTE and an outpatient anticoagulant prescription following prostate cancer diagnosis. Men were followed to identify cases of recurrent VTE, and hospitalized major bleeding. We calculated adjusted hazard ratios (HRs) with 95% confidence intervals (CIs) to quantify the association between anticoagulation duration (reference ≤ 3 months) and recurrent VTE using Cox regression. We estimated 1-year cumulative incidences of major bleedings from anticoagulation initiation.

**Results:**

The outpatient anticoagulation prescribed was parenteral (64%), direct oral anticoagulant (31%), and vitamin K antagonist (20%). Median duration of anticoagulation was 7 months. Adjusted HRs (95% CI) for off-treatment recurrent pulmonary embolism (PE) were 0.32 (0.09–1.15) for > 3–6 months’ duration, 0.21 (0.06–0.69) for > 6–9 months and 0.16 (0.05–0.55) for > 9 months; corresponding HRs for deep vein thrombosis (DVT) were 0.67 (0.27–1.66), 0.80 (0.31–2.07), and 1.19 (0.47–3.02). One-year cumulative incidences of intracranial, gastrointestinal and urogenital bleeding were 0.9%, 1.7%, 3.0% during treatment, and 1.2%, 0.9%, 1.6% after treatment cessation.

**Conclusion:**

The greatest possible benefit in reducing recurrent VTE risk occurred with > 9 months anticoagulation for PE and > 3–6 months for DVT, but larger studies are needed to confirm this. Risks of major bleeding were low overall.

**Supplementary Information:**

The online version contains supplementary material available at 10.1186/s12894-022-00967-z.

## Introduction

Venous thromboembolism (VTE) is one of the leading causes of death in patients with cancer after disease progression [1–3], and around 15–20% of all patients with VTE have cancer [1, 4, 5]. Prostate cancer is the most commonly diagnosed cancer in men aged ≥ 55 years worldwide [6]. Although prostate cancer is associated with a low risk of VTE relative to other cancer types, the risk of VTE has been estimated to be three-fold higher in men with prostate cancer than among men of the same age without cancer in the general population [7]. Owing to the high prevalence of prostate cancer, VTE is commonly seen among affected men in clinical practice [1], hence the importance of prescribing effective agents for treatment and secondary prophylaxis.

For the majority of patients with cancer-associated thrombosis, clinical guidelines recommend long-term anticoagulant therapy—usually with low-molecular weight heparin (LMWH) or a direct oral anticoagulant (DOAC)—to help prevent VTE recurrence. Some guidelines advocate at least 3 months of anticoagulant therapy [8], and others recommend 6 months [9] or a minimum of 6 months [10, 11], and there is currently no consensus regarding therapy beyond 6 months. The pivotal clinical trials that shaped guideline development were limited to 3–6 months duration [12], and observational data on the topic are limited. Determining the optimal duration of anticoagulant therapy requires balancing the reduced risk of VTE recurrence against the increased risk of bleeding, which is also raised in patients with cancer. Whether prolonged anticoagulation beyond 6 months affords a favourable benefit–risk ratio is a question that remains unanswered.

The aim of this study was to evaluate the type and duration of long-term anticoagulant therapy in men with prostate cancer and VTE, the association between duration of treatment and risk of VTE recurrence after treatment cessation, and risk of major bleedings on- and off-treatment. Of note is that we refrain from using the term 'cancer-associated VTE' because of the difficulty in determining the true likely cause of the thrombosis using our study design that used secondary collected data.

## Methods

### Data source

We used data from the Prostate Cancer data Base Sweden (PCBaSe) 4.0, a research database comprising information from the National Prostate Cancer Register in Sweden and other linked national health registers. Details of PCBaSe have been published previously [13, 14]. Briefly, the database contains information on 180,000 men diagnosed with prostate cancer between 1998 and 2016 and captures 98% of all newly diagnosed cases of prostate cancer in Sweden since 1998. For this study, we used data from the National Patient Registry [15], which captures hospital inpatient/outpatient diagnoses using International Classification of Diseases (ICD-10) codes (with a look-back period to 1998), and the Prescribed Drug Registry (with a look-back period to 2005) [16], which records medications dispensed from all Swedish pharmacies using Anatomical Therapeutic Classification (ATC) codes. We also obtained data from the Swedish longitudinal integration database for health insurance and labour market Register for information on education level and marital status, and data from the Registries of Immigration and Emigration. The study protocol was approved by the Research Ethics Authority, Uppsala (Dnr 2019-01319).

### Identification of men with prostate cancer and VTE

**Fig. 1:**
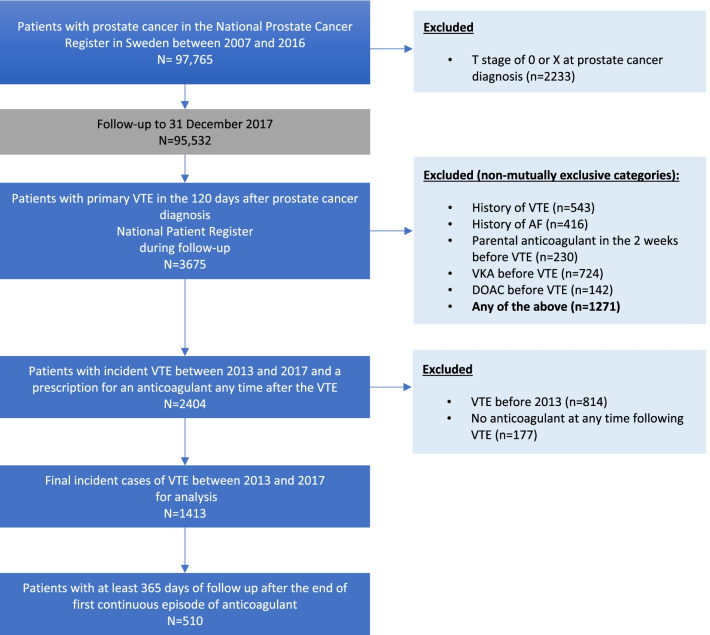
Identification of men with prostate cancer and VTE treated with anticoagulant therapy. *ICD-10 codes I82, I801, I802, I803, I809 as main diagnosis. AF, arial fibrillation; DOAC, direct oral anticoagulant; VKA, vitamin K antagonist; VTE, venous thromboembolism

A flowchart depicting the identification of men with prostate cancer and VTE is shown in Fig. 1. The source population included all men in PCBaSe 4.0 with a first diagnosis of prostate cancer between 1 January 2007 (about 18 months after the Prescribed Drug Registry became available) and 31 December 2016, with at least 1 year of observational follow-up. From this source population, we included all men with a first inpatient/outpatient diagnosis of VTE (index VTE; ICD-10 codes I82, I801, I802, I803, I809) recorded as a primary diagnosis in the National Patient Registry, and with a prescription for an anticoagulant in the Prescribed Drug Registry any time after the index VTE (N = 2404). We excluded men with either a past record of VTE (in any position, not restricted to the main diagnosis) or atrial fibrillation and those with a prescription for oral anticoagulant treatment any time before the index VTE, or for parental anticoagulant treatment in the 2 weeks before the index VTE (see Additional file 1: Supplementary Table 1 for codes). For our analyses, we retained only men whose index VTE was recorded between 2013 and 2017, to cover the period from which DOACs were approved in Sweden.

### Anticoagulant treatment for VTE

We classified the type of anticoagulant(s) dispensed during the 4 weeks after the index VTE date as either parenteral anticoagulant, vitamin K antagonist (VKA) or DOAC; men could have been prescribed more than one type of anticoagulant during this period, either as a bridge between parenteral and oral agents or because they switched between drug class. The duration of an individual prescription was calculated based on all dispensed prescriptions any time after index VTE using information on pack size and the numbers of packs dispensed. Total anticoagulant exposure was determined by summing the lengths of individual consecutive prescriptions. A gap of more than 30% of supplied days was considered to represent the cessation of anticoagulant exposure. To validate exposure in relation to recurrent VTE events, the start and end dates of anticoagulation prescription records were manually reviewed by one investigator (BF) to increase the level of certainty that the event occurred after the cessation of anticoagulation. We categorized exposure duration as ≤ 3 months, > 3 to 6 months, > 6 to 9 months and > 9 months irrespective of anticoagulant type.

### Co-variates

We obtained information on sociodemographics (including educational and marital status), comorbidities and comedications before the index VTE. The Charlson comorbidity index was determined at the time of prostate cancer diagnosis.

### Recurrent VTE

To identify cases of off-treatment recurrent VTE, we followed-up men from the date that their anticoagulation treatment ended until the earliest of the following: the first subsequent entry of a VTE diagnosis as the main diagnosis, death, the date of emigration, or the end of available follow-up (data lock point) in PCBaSe (31 December 2017). For this analysis, the study population was restricted to men whose treatment cessation occurred at least 1-year before the data-lock date to ensure adequate potential follow-up time to identify VTE events.

### Major bleeding

We performed separate follow-ups to identify cases of major bleeding as the main cause of hospitalisation, one for each bleeding type (intracranial, gastrointestinal, urogenital or ‘other bleeding’ (see Additional file 1: Supplementary Table 2 for codes). We followed-up men from the start of anticoagulation therapy until the earliest of the following: the first subsequent entry of the major bleeding, death, the date of emigration, or the end of follow-up. We recorded whether the bleeding event occurred on-treatment or off-treatment (after ending of the anticoagulant treatment).

### Statistical analysis

We summarised sociodemographics, clinical characteristics and duration of anticoagulant exposure using frequency counts and percentages for categorical variables, and with medians with interquartile range (IQR) for continuous variables. One-year cumulative incidences of recurrent VTE and major bleeding were calculated by dividing the number of incident cases during the first year of follow-up by the number of men at the start of follow-up (patients were not censored at death or low-to follow-up), with 95% confidence intervals computed using the binominal distribution. For major bleeding, cumulative incidence estimates were also stratified by type of bleeding. Incidence rates of recurrent VTE and major bleeding over the whole period of observation were calculated as the number of VTE/major bleeding events divided by the total person-years of follow-up, with 95% confidence intervals (CIs) determined using the Poisson distribution. We used Cox proportional hazards regression models to compute hazard ratios (HRs) with 95% CIs for associations between duration of anticoagulant exposure and off-treatment recurrent VTE adjusted for confounders and using ≤ 3 months duration as the reference group. We present age-adjusted HRs based on the finding that no additional variable made any notable change to the risk estimate when included into the model. SAS version 9.4 was used for all analyses.

## Results

### Baseline characteristics of patients with incident VTE

A total of 1413 men with prostate cancer experienced a VTE and were dispensed anticoagulant therapy from a pharmacy: 50% (700/1413) had a pulmonary embolism (PE), of which 68% were hospitalised, and 45% (640/1413) had a deep vein thrombosis (DVT), of which 12% were hospitalised (5%, 73/1413 had a non-specified VTE). Clinical and sociodemographic characteristics are shown in Additional file 1: Supplementary Tables 3–5. Median age was 73 years (IQR 68–79) at VTE diagnosis. Median time from prostate cancer diagnosis to VTE diagnosis was 3 years (IQR 1–6). Approximately 20% of patients experienced their VTE within 1 year after prostate cancer diagnosis. Common comorbidities included hypertension (67%), cerebrovascular disease (10%), diabetes (14%) and hyperlipidaemia (12%), and just over 10% of patients had a history of urogenital bleeding. Commonly used medications at baseline (any time before index VTE) included antihypertensives (67%), statins (35%) and antiplatelets (30%). No major differences were seen in the clinical profiles of men with DVT vs. those with PE.

### Anticoagulation

**Fig. 2:**
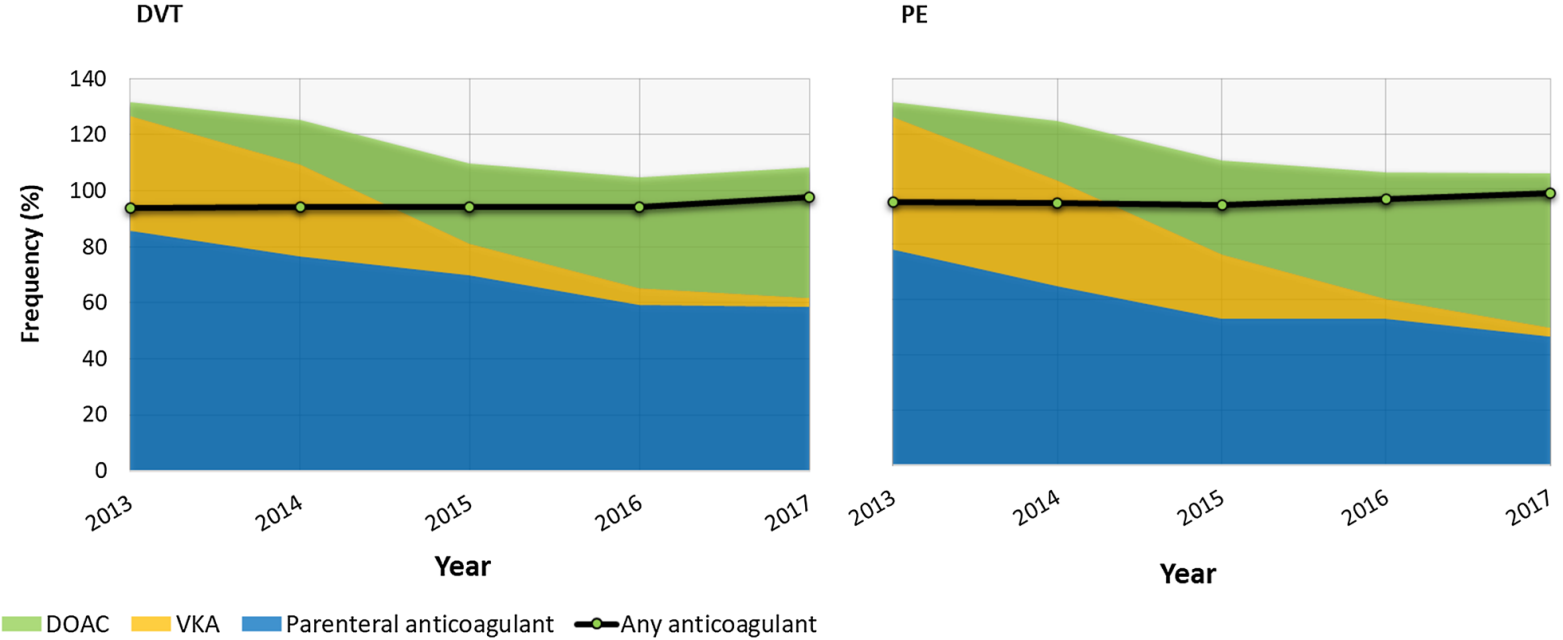
Type of anticoagulant(s) prescribed during the 4 weeks after the index VTE among the 1413 men with prostate cancer and VTE (2013–2017) stratified by type of VTE (DVT/PE). *Note*: Men were eligible to have received more than one type of anticoagulant treatment during this 4-week time period. In total, an outpatient prescription for an anticoagulant during the 4 weeks after the index VTE was issued to 95.5% of men, with 4.5% of me issued an anticoagulant prescription after 4 weeks. DOAC, direct oral anticoagulant; DVT, deep vein thrombosis; PE, pulmonary embolism; VKA, vitamin K antagonist; VTE, venous thromboembolism

The vast majority (96%) of men started their outpatient anticoagulant therapy within the 4 weeks after their VTE diagnosis. Men received their first prescription at a median of 2 days after their VTE (range 1–6 days). Almost two-thirds (64%) were prescribed parenteral anticoagulation, 31% a DOAC and 20% a VKA (Fig. 2). The substantial decline between 2013 and 2017 in the use of parental anticoagulation (83% to 53%) and VKAs (45% to 4%) corresponded with a substantial increase in DOAC use (5% to 51%). The median duration of use was 7 months (IQR 3–13). Fifty seven per cent of men were prescribed > 6 months’ anticoagulation, and a quarter were prescribed > 1 year of anticoagulation (Additional file 1: Supplementary Table 6). In general, anticoagulation was longer for PE (median 8 months, IQR 5–15) than for DVT (median 6 months, IQR 3–9). Over two-thirds (67%) of men treated for PE received outpatient anticoagulation for more than > 6 months, with over a third (35%) treated for > 1 year (Additional file 1: Supplementary Table 6).

### Recurrent VTE and major bleeding

Five hundred and ten men were included in the analysis of recurrent VTE. Incidence rates of recurrent VTE over the whole of follow-up are shown in Additional file 1: Supplementary Table 7a. The incidence rate of recurrent VTE per 100 person-years was 19.2 (95% CI 11.2–30.8) in men with ≤ 3 months’ therapy, 13.1 per 100 person-years (95% CI 7.3–21.5) for > 3–6 months, 14.7 per 100 person-years (95% CI 8.9–22.6) for > 6–9 months, and 15.0 per 100 person-years (95% CI 8.9–23.7) for > 9 months. As shown in Table 1, compared with men with ≤ 3 months’ anticoagulation for the index PE, adjusted HRs for recurrent VTE were 0.32 (95% CI 0.09–1.12) for > 3–6 months therapy, 0.26 (95% CI 0.08–0.82) for > 6–9 months, and 0.18 (95% CI 0.15–0.60) for > 9 months. Corresponding HRs relating to treatment of the index DVT were 0.67 (95% CI 0.27–1.66) for > 3–6 months, 0.80 (95% CI 0.31–2.07) for > 6–9 months, and 1.19 (95% CI 0.47–3.02) for > 9 months.

**Table 1:** HRs (95% CIs) for recurrent VTE after the cessation of anticoagulation for the index VTE by duration of use in men diagnosed with prostate cancer between 2007 and 2016 and followed until December 31, 2017, and registered in the NPCR of Sweden

Duration of anticoagulant therapy	DVT			PE			VTE^*^		
	Incident cases/person-years	Crude RR (95% CI)	Adjusted RR^†^ (95% CI)	Incident cases/person-years	Crude RR (95% CI)	Adjusted RR^†^ (95% CI)	Incident cases/person-years	Crude RR (95% CI)	Adjusted RR^†^ (95% CI)
≤ 3 months N = 102	11/68	1.00	1.00	5/13	1.00	1.00	17^**†**^/88	1.00	1.00
> 3 to 6 months N = 127	9/75	0.74 (0.31–1.79)	0.71 (0.29–1.74)	6/35	0.47 (0.14–1.55)	0.32 (0.09–1.12)	15/115	0.68 (0.34–1.37)	0.66 (0.33–1.33)
> 6 to 9 months N = 150	9/60	0.93 (0.39–2.24)	0.99 (0.41–2.43)	11/68	0.45 (0.16–1.29)	0.26 (0.08–0.82)	20/137	0.77 (0.40–1.46)	0.76 (0.40–1.45)
> 9 months N = 131	9/39	1.41 (0.58–3.40)	1.39 (0.56–3.44)	8/75	0.30 (0.10–0.90)	0.18 (0.05–0.60)	18^**†/**^120	0.78 (0.40–1.52)	0.74 (0.38–1.44)

CI, confidence interval; DVT, deep vein thrombosis; DOAC, direct oral anticoagulant; HR, hazard ratio; LMWH, low-molecular weight heparin; NPCR, National Prostate Cancer Register; NSAID, non-steroidal anti-inflammatory drug; PE, pulmonary embolism; VKA, vitamin K antagonist; VTE, venous thromboembolism

^*^Includes 73 men with VTE not specified as either DVT/PE, either ICD-10 I809 or I82)

^†^Adjusted for age, time from prostate cancer to VTE diagnosis, and the following comorbidities before the index VTE: cardiovascular disease, hypertension, diabetes, major bleeding, cerebrovascular disease, and use of statins or NSAIDS before the index VTE

^**†**^One case was classified as neither DVT or PE, but as 'other VTE'

Analysis included 510 men with VTE and at least 1 year of follow-up after the end of therapy). Anticoagulants included LMWH, VKAs and DOACs

**Fig. 3:**
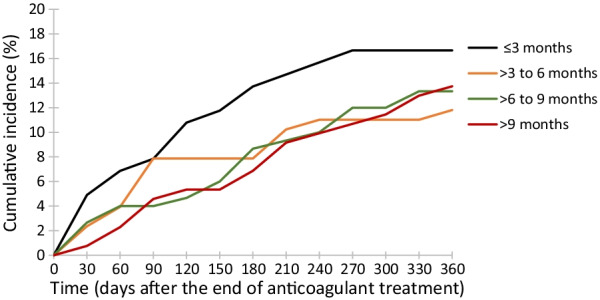
Cumulative incidence (%) of recurrent VTE after the cessation of anticoagulant treatment by duration of therapy and time since cessation of therapy. *Note*: Analysis included 510 men with VTE and at least 1 year of follow-up after the cessation of therapy. VTE, venous thromboembolism

One-year cumulative incidences of recurrent VTE after the cessation of anticoagulant therapy were 17% for ≤ 3 months, 12% for > 3–6 months, 13% for > 6–9 months, and 14% for > 9 months (Additional file 1: Supplementary Table 8). Cumulative incidence of recurrent VTE was highest in men with ≤ 3 months anticoagulation at all timepoints during the year after treatment cessation. Cumulative incidences at 60 days after anticoagulation cessation were 7% for men who received ≤ 3 months’ duration, 4% for > 3–6 months or > 6–9 months’ therapy, and 2% for > 9 months’ therapy (Fig. 3); corresponding estimates at 180 days were 14%, 8%, 9%, and 7%, respectively, and at 360 days, they were 17%, 12%, 13%, and 14%, respectively.

**Fig. 4:**
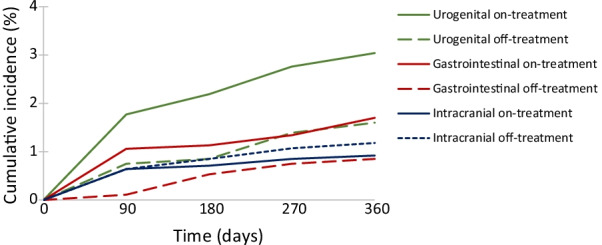
Cumulative incidence (%) of on-treatment and off-treatment major bleeding of anticoagulant therapy. *Note*: On-treatment analysis included all 1413 men with VTE, and off-treatment analysis include 936 patients. VTE, venous thromboembolism

Incidence rates of major bleeding rates were low; rates over the whole of follow-up are shown in Additional file 1: Supplementary Table 7b. One-year cumulative incidences for urogenital bleeding were 3.0% (on-treatment) vs. 1.6% (off-treatment), for gastrointestinal bleeding were 1.7% (on-treatment) vs. 0.9% (off-treatment), and for intracranial bleeding were 0.9% (on-treatment) vs. 1.2% (off-treatment) (Fig. 4; Additional file 1: Supplementary Table 9). The cumulative incidence of most types of major bleeding increased most rapidly over the first 90 days and increased more steadily thereafter (Fig. 4).

## Discussion

In this large population-based study of men with prostate cancer in Sweden, the average duration of outpatient anticoagulant therapy for the treatment and secondary prophylaxis of VTE was 7 months; however, duration of treatment varied between patients. A fifth of men received anticoagulation for ≤ 3 months, while a quarter received ≥ 1 year duration of therapy. For cases of PE, anticoagulation was, on average, prescribed for a longer period. A reduced risk of recurrent VTE was seen among patients prescribed > 3–6 months therapy (vs ≤ 3 months therapy). Although the point estimates suggest that > 9 months treatment might afford the greatest benefit for PE, and that > 3–6 months might afford the greatest benefit for DVT; the CIs overlapped with those for other duration categories, thus larger, more powered studies are needed to confirm this. Risks of major bleeding were low both on- and off-treatment, especially for intracranial bleeding. The differences between the on-treatment and the slightly lower off-treatment bleeding risks was most apparent for urogenital bleeding (3.0% vs. 1.6%).

Previous studies on this topic included patients across several cancer sites with different anticoagulation requirements and have evaluated VTE risks using different definitions. The average duration of anticoagulation among our study cohort (7 months) is similar to that seen among cancer patients in the international RIETE registry (6 months) [17]. In US claims database studies, the average length of anticoagulation for VTE has been higher. Between 2007 and 2011, Kaatz et al. [18] found that cancer patients were anticoagulated for VTE (mostly warfarin) for an average of 10 months, while for the period 2007–2014, Khorana et al. [19] reported a median duration of 8 months for warfarin/rivaroxaban therapy among patients with cancer. We observed a significant increase in DOAC use for VTE among men in our study (reaching 51% in 2017), with a corresponding decline in use of VKAs (4% in 2017) and of parenteral anticoagulants (53% in 2017), albeit the latter was still used by over half of men long-term. This rise in DOAC use is consistent with temporal trends in other cancer populations, and reflect the current guideline recommendations [20]. It is important to note that our data did not enable us to determine whether a recurrent VTE event was ‘cancer-associated’, nor was this the aim of the study—our focus was on all men with prostate cancer who were diagnosed with a VTE at any time point following cancer diagnosis, irrespective of treatment. In men with prostate cancer, it is challenging to determine whether a VTE event is cancer-associated because this type of cancer can have slow progression, and patients may require different treatments depending on disease stage; thrombotic activity could therefore be triggered at different times in different patients. Furthermore, it is noteworthy that only about 20% of men in our study were diagnosed with a VTE within a year of cancer diagnosis.

Owing to advances in early screening and treatment, life expectancy of men with prostate cancer can be relatively long when compared with other cancer types, and timely diagnosis and treatment of VTE in these men is important because it can impact prognosis and survival. Yet, anticoagulant treatment decisions are challenging because these patients are characteristically of older age and with comorbidities that may increase thromboembolism and bleeding risks. Our findings reflect the adoption of an individualised approach to treating VTE in this heterogenous population. Despite the clinical guidance for at least 3-months’ anticoagulation for VTE in patients with cancer, approximately 1 in 5 men in our study were treated for only 3 months or less, highlighting the importance of analysing observational data to reveal actual prescribing practices. Reasons for this potential under-prescribing may be due to a range of patient- and cancer-related factors, as well as caution by physicians in relation to bleeding risks, and requires investigation in future studies. Notwithstanding the necessity for personalized treatment approaches, a higher risk of recurrent VTE was seen in men treated with ≤ 3 months’ anticoagulation vs longer durations. This is consistent with findings among cancer patients in the claims database study by Khorana et al. [21], and among studies of patients without cancer from trials and/or observational studies [22, 23]. Furthermore, the 1-year 17% VTE recurrence rate among men who stopped anticoagulation after ≤ 3 months in our study is unacceptably high and warrants the attention of clinicians. The small possible increase in recurrent DVT risk observed in men treated for > 9 months is likely to be an artefact from an inherent increased in this subset of patients. Although risks of major bleeding were low during both the on- and off-treatment periods, the risk of urogenital bleeding (which is not unexpected considering the underlying diagnosis of prostate cancer) was higher during the on-treatment period. This is important to note because it could potentially result in suspension of treatment. Accordingly, it would be beneficial for patients and physicians to be vigilant about the potential for urogenital bleeding, especially during the anticoagulation period.

A major strength of our study is the focus on a single cancer type. Selection bias was minimized because the cohort included unselected men with prostate cancer of different stages/grades, across all ages and including those with comorbidities. Sweden has a tax-funded public healthcare system. Data completeness in the Swedish Prescribed Drug Register is virtually complete because Swedish pharmacies are required to participate by law. We therefore captured treatment patterns in virtually all men with prostate cancer and VTE in the country, none of whom were lost to follow-up. Although drugs issued in hospital are not captured in PCBaSe, we were interested in long-term anticoagulation, where the minimum recommended duration is 3 months. Furthermore, the proportion of men hospitalised for their VTE was low (68% for PE and 12% for DVT). We included both inpatient and outpatient VTE events, based on previous knowledge that a high proportion of VTE events in cancer patients occur in the ambulatory setting [5]. The main study limitation is the likelihood of residual confounding from factors such as VTE severity. Other factors that influence VTE risk, such as cancer stage/grade, cancer treatment (including surgery), age and comorbidity profile, all of which can affect VTE risk [24, 25], may also have influenced treatment decisions, with the duration of treatment dependent on the individual’s risk profile. We were unable to validate the VTE diagnoses as the results of imaging procedures are not routinely recorded in the Patient Register. Furthermore, information on the accuracy of the ICD-10 codes for VTE used in clinical practice across Sweden are lacking, and the uncertain accuracy of routinely recorded information on cause of death in the Swedish Cause of Death Registry prevented the ascertainment of fatal VTE events. Another study limitation was the inability to distinguish between incidental and symptomatic VTE events (and therefore evaluate any differences in anticoagulant treatment patterns between the two) because these will have been recorded in the same way (i.e. main diagnosis coded entries). Additionally, anticoagulant duration may have been misclassified because information on adherence was unavailable, and this may have impacted on our VTE recurrence/bleeding analyses. Lastly, some of our analyses may have been underpowered to detect significant differences between anticoagulant duration categories.

In conclusion, our study provides useful information on the benefits and risks of anticoagulation therapy beyond 6 months’ duration for VTE in men with prostate cancer, on which there is currently no consensus. While our results suggest the greatest potential for the reducing VTE recurrence occurs with > 9 months treatment for PE and > 3–6 months for DVT, larger, more powered studies from real-world clinical practice are needed to confirm this. Risks of major bleeding were low overall, although the higher risks of urogenital bleeding seen during the anticoagulation period warrants vigilance by patients and physicians. Further epidemiological studies are needed to confirm/refute our findings and help guide clinical decision making in routine practice. Other beneficial areas for further research in this field include the effects of specific anticoagulants on relevant clinical outcomes and gaining a better understanding of the prescribing choices.

## Supplementary Information

**Additional file 1: Supplementary Table 1.** ATC codes for anticoagulant drugs. **Supplementary Table 2.** ICD-10 codes for major bleeding outcomes. **Supplementary Table 3.** Clinical and sociodemographic characteristics of the 1413 men with prostate cancer and VTE (2013–2017) at the time of prostate cancer diagnosis. **Supplementary Table 4.** Charlson comorbidity index, and distribution of comorbidities, among the 1413 men with prostate cancer and VTE (2013–2017). **Supplementary Table 5.** Frequency distribution of prescribed drugs any time before the VTE among the 1413 men with prostate cancer and VTE (2013–2017). **Supplementary Table 6.** Duration of anticoagulation by type of VTE. **Supplementary Table 7a.** Incidence rates of recurrent VTE per 100 person-years after the cessation of anticoagulant therapy by duration of therapy. **Supplementary Table 7b.** Incidence rates of ‘on-treatment’ and ‘off-treatment’ major bleeding events per 100 person-years. **Supplementary Table 8.** Cumulative incidence (%) of recurrent VTE after the cessation of anticoagulant therapy according to the duration of anticoagulant therapy for the index VTE. **Supplementary Table 9.** Cumulative incidence (%) of on-treatment and off-treatment major bleeding.

## Data Availability

The datasets analysed during the current study are available from the corresponding author on reasonable request.

## Abbreviations

ATC: Anatomical Therapeutic Classification
CI: Confidence interval
DOAC: Direct oral anticoagulant
HR: Hazard ratio
IQR: Inter-quartile range
LMWH: Low-molecular weight heparin
PCBaSe: Prostate Cancer data Base Sweden
VKA: Vitamin K antagonist
VTE: Venous thromboembolism
