# Supplementary material for: Population-based study of long-term anticoagulation for treatment and secondary prophylaxis of venous thromboembolism in men with prostate cancer in Sweden
Source: BMC Urol. 2022 Feb 2;22:15. doi: 10.1186/s12894-022-00967-z (PMC8809008; doi:10.1186/s12894-022-00967-z)
Supplement: Supplementary file 1 — Additional file 1: Supplementary Table 1. ATC codes for anticoagulant drugs. Supplementary Table 2. ICD-10 codes for major bleeding outcomes. Supplementary Table 3. Clinical and sociodemographic characteristics of the 1413 men with prostate cancer and VTE (2013–2017) at the time of prostate cancer diagnosis. Supplementary Table 4. Charlson comorbidity index, and distribution of comorbidities, among the 1413 men with prostate cancer and VTE (2013–2017). Supplementary Table 5. Frequency distribution of prescribed drugs any time before the VTE among the 1413 men with prostate cancer and VTE (2013–2017). Supplementary Table 6. Duration of anticoagulation by type of VTE. Supplementary Table 7a. Incidence rates of recurrent VTE per 100 person-years after the cessation of anticoagulant therapy by duration of therapy. Supplementary Table 7b. Incidence rates of ‘on-treatment’ and ‘off-treatment’ major bleeding events per 100 person-years. Supplementary Table 8. Cumulative incidence (%) of recurrent VTE after the cessation of anticoagulant therapy according to the duration of anticoagulant therapy for the index VTE. Supplementary Table 9. Cumulative incidence (%) of on-treatment and off-treatment major bleeding. [file 12894_2022_967_MOESM1_ESM.pdf]

Supplement

Supplementary Table 1. ATC codes for anticoagulant drugs.

Class	Drug name	ATC classification code
**1. Parenteral anticoagulants**	Heparin	B01AB01
	Enoxaparin	B01AB05
	Dalteparin	B01AB04
	Nadroparin	B01AB06
	Tinzaparin	B01AB10
	Reviparin	B01AB08
	Parnaparin	B01AB07
	Bemiparin	B01AB12
	Fondaparinux	B01AX05
	Danaparoid	B01AB09
	Bivalirudin	B01AE06
	Argatroban	B01AE03
**2. Vitamin K antagonists**	Warfarin	B01AA03
	Phenprocoumon	B01AA04
	Acenocoumarol	B01AA07
	Dicoumarol	B01AA01
	Tioclomarol	B01AA11
	Ethyl biscoumacetate	B01AA08
	Fluindione	B01AA12
	Phenindione	B01AA02
	Chlorindione	B01AA09
	Diphenadione	B01AA10
**3. Non-vitamin K oral anticoagulants (NOACs)**	Rivaroxaban (Xarelto)	B01AF01
	Dabigatran (Pradaxa)	B01AE07
	Apixaban (Eliquis)	B01AF02
	Edoxaban (Savaysa/Lixiana)	B01AF03

ATC, Anatomical Therapeutic Chemical

Supplementary Table 2. ICD-10 codes for major bleeding outcomes.

Condition	ICD-10 or Swedish procedure codes
Intracranial bleeding	I60-62, S064, S065, S066
Gastrointestinal bleeding	I850, I983, K226, K250, K252, K254, K256, K260, K262, K264, K266, K270, K272, K274, K276, K280, K284, K286, K290, K625, K661, K920, K921, K922
Urogenital bleeding	N02, R319, N95
Other bleeding	H431, R04, R58, D629, procedure code DR029

ICD, international Classification of Diseases

Supplementary Table 3. Clinical and sociodemographic characteristics of the 1413 men with prostate cancer and VTE (2013–2017) at the time of prostate cancer diagnosis.

	DVT N=640	PE N=700	VTE* N=1413
**Age at prostate cancer diagnosis (years), median (IQR)**	69.0 (64.0–75.0)	70.0 (65.0–75.0)	70.0 (64.0–75.0)
**Prostate cancer risk group***			
Low-intermediate	354 (55.3)	380 (54.3)	777 (55.0)
High-locally metastatic	207 (32.3)	203 (29.0)	428 (30.3)
Distant metastases	70 (10.9)	110 (15.7)	192 (13.6)
Missing	9 (1.4)	7 (1.0)	16 (1.1)
**TNM stage**			
T1	299 (46.7)	348 (49.7)	684 (48.4)
T2	186 (29.1)	206 (29.4)	416 (29.4)
T3	126 (19.7)	124 (17.7)	260 (18.4)
T4	29 (4.5)	22 (3.1)	53 (3.8)
N0	122 (19.1)	151 21.6)	283 (20.0)
N1	35 (5.5)	35 (5.0)	75 (5.3)
NX	481 (75.2)	514 (73.4)	1053 (74.5)
M0	392 (61.3)	422 (60.3)	858 (60.7)
M1	45 (7.0)	84 (12.0)	139 (9.8)
MX	201 (31.4)	194 (27.7)	414 (29.3)
**PSA at prostate cancer diagnosis (mg/l)**			
<3	23 (3.6)	24 (3.4)	53 (3.8)
3–10	309 (48.3)	370 (52.9)	717 (50.7)
11–50	197 (30.8)	198 (28.3)	412 (29.2)
≥51	99 (15.5)	99 (14.1)	210 (14.9)
Missing	12 (1.9)	9 (1.3)	21 (1.5)
**Time from prostate cancer diagnosis to VTE diagnosis (years)**			
≤1	123 (19.2)	144 (20.6)	280 (19.8)
>1–2	81 (12.7)	96 (13.7)	187 (13.2)
>2–3	67 (10.5)	108 (15.4)	186 (13.2)
>3	369 (57.7)	352 (50.3)	760 (53.8)
Median (IQR)	3.9 (1.3–6.1)	3.0 (1.4–5.5)	3.3 (1.4–5.7)
**Median age (IQR) at incident VTE diagnosis (years)**	73.0 (68.0–79.0)	73.0 (68.0–79.0)	73.0 (68.0–79.0)
**Hospitalised for VTE**	77 (12.0)	478 (68.3)	576 (40.8)
**Educational level**			
Low (<9 years)	213 (33.3)	254 (36.3)	487 (34.5)
Middle (9–12 years)	264 (41.3)	266 (38.0)	560 (39.6)
High (>12 years/university)	160 (25.0)	171 (24.4)	353 (25.0)
Missing	3 (0.5)	9 (1.3)	13 (0.9)
**Marital status**			
Married	413 (64.5)	472 (67.4)	933 (66.0)
Unmarried	75 (11.7)	79 (11.3)	165 (11.7)
Divorced	109 (17.0)	95 (13.6)	212 (15.0)
Widower	43 (6.7)	53 (7.6)	102 (8.2)

Data are n (%) unless otherwise stated.
*Includes 73 men with VTE not specified as either DVT/PE, either ICD-10 I809 or I82).

DVT, deep vein thrombosis; IQR, interquartile range; PE, pulmonary embolism; PSA, prostate specific antigen; VTE, venous thromboembolism

Supplementary Table 4. Charlson comorbidity index, and distribution of comorbidities, among the 1413 men with prostate cancer and VTE (2013–2017).

	DVT N=640	PE N=700	VTE* N=1413
**Charlson comorbidity index (at prostate cancer diagnosis)**			
0	527 (82.3)	551 (78.7)	1137 (80.5)
1	56 (8.8)	71 (10.1)	135 (9.6)
2	27 (4.2)	44 (6.3)	76 (5.4)
3	15 (2.3)	21 (3.0)	37 (2.6)
≥4	15 (2.3)	13 (1.9)	28 (2.0)
Valvular heart disease	14 (2.2)	19(2.7)	35 (2.5)
Cardiovascular disease	125 (19.5)	144 (20.6)	281 (19.9)
Chronic heart failure	42 (6.6)	48 (6.9)	92 (6.5)
Coronary artery disease	86 (13.4)	96 (13.7)	191 (13.5)
Unstable angina pectoris	25 (3.9)	27 (3.9)	52 (3.7)
Angina pectoris	62 (9.7)	68 (9.7)	134 (9.5)
Peripheral arterial disease	36 (5.6)	41 (5.9)	81 (5.7)
Myocardial infarction	60 (9.4)	68 (9.7)	134 (9.5)
Hypertension			
ICD-10	261 (40.8)	303 (43.3)	590 (41.8)
ICD-10 or ATC Classification code	420 (65.6)	482 (68.9)	952 (67.4)
Diabetes mellitus			
ICD-10	79 (12.3)	75 (10.7)	166 (11.7)
ICD-10 or ATC	98 (15.3)	88 (12.6)	200 (14.2)
Liver disease	8 (1.3)	6 (0.9)	14 (1.0)
Hyperlipidemia	74 (11.6)	88 (12.6)	169 (12.0)
Obstructive sleep apnea	16 (2.5)	15 (2.1)	31 (2.2)
COPD	38 (5.9)	51 (7.3)	93 (6.6)
Diabetic retinopathy	15 (2.3)	7 (1.0)	23 (1.6)
Acute renal failure	14 (2.2)	22 (3.1)	39 (2.8)
Peripheral systemic embolism	4 (0.6)	4 (0.1)	6 (0.4)
Limb ischemia	13 (2.0)	11 (1.6)	25 (1.8)
Limb ulcer	4 (0.6)	7 (1.0)	11 (0.8)
Cerebrovascular disease	65 (10.2)	73 (10.4)	143 (10.1)
Ischaemic stroke	42 (6.6)	45 (6.4)	89 (6.3)
Stroke NOS	16 (2.5)	10 (1.4)	26 (1.8)
TIA	18 (2.8)	20 (2.9)	40 (2.8)
Hemorrhagic stroke	11 (1.7)	19 (2.7)	31 (2.2)
Any major bleeding	110 (17.2)	118 (16.9)	238 (16.8)
Intracranial bleeding	15 (2.3)	25 (3.6)	41 (2.9)
Upper GI bleeding	30 (4.7)	31 (4.4)	61 (4.3)
Lower GI bleeding	5 (0.8)	6 (0.9)	13 (0.9)
Urogenital bleeding	69 (10.8)	72 (10.3)	150 (10.6)

Data are n (%).

*Includes 73 men with VTE not specified as either DVT/PE, either ICD-10 I809 or I82).

Note: comorbidities are any time before the index VTE.

ATC, ATC, Anatomical Therapeutic Chemical; COPD, Chronic obstructive pulmonary disease; DVT, deep vein thrombosis; ICD, International Classification of Diseases; NOS, not otherwise specified; PE, pulmonary embolism; TIA, transient ischaemic attack; VTE, venous thromboembolism

Supplementary Table 5. Frequency distribution of prescribed drugs any time before the VTE among the 1413 men with prostate cancer and VTE (2013–2017).

	**DVT N=640**		**PE N=700**		**VTE* N=1413**	
	**n**	**%**	**n**	**%**	**n**	**%**
Antiarrhythmics	2	0.3	1	0.1	3	0.2
Statins	222	34.7	254	36.3	500	35.4
Antihypertensive drugs	415	64.8	480	68.6	945	66.9
Beta-blockers	225	35.2	254	36.3	501	35.5
Calcium-channel blockers	208	32.5	249	35.6	476	33.7
ACE inhibitors	237	37.0	266	38.0	537	38.0
Angiotensin II receptor blockers	149	23.3	185	26.4	353	25.0
Diuretics	212	33.1	252	36.0	484	34.3
Antidiabetics	78	12.2	77	11.0	169	12.0
NSAID	454	70.9	509	72.7	1019	72.1
**Antiplatelet drugs** acetyl acid	181	28.3	224	32.0	421	29.8
**Parenteral anticoagulant**	219	34.2	211	30.1	444	31.4

*Includes 73 men with VTE not specified as either DVT/PE, either ICD-10 I809 or I82).
ACE, angiotensin-converting enzyme; DVT, deep vein thrombosis; NSAID, non-steroidal anti-inflammatory drug; PE, pulmonary embolism; VTE, venous thromboembolism

Supplementary Table 6. Duration of anticoagulation by type of VTE.

Duration of anticoagulation (days)	DVT (N=640)	PE (N=700)	VTE* N=1413)
≤3 months	178 (27.8)	100 (14.3)	298 (21.1)
>3 to 6 months	168 (26.3)	128 (18.3)	311 (22.0)
>6 to 9 months	132 (20.6)	151 (21.6)	297 (21.0)
>9 months	162 (25.3)	321 (45.9)	507 (35.9)
Median (IQR)	168 (85–278)	246 (157–462)	205 (103–376)

Data are n (%)

*Includes 73 men with VTE not specified as either DVT/PE, either ICD-10 I809 or I82).

DVT, deep vein thrombosis; PE, pulmonary embolism; VTE, venous thromboembolism

Supplementary Table 7a. Incidence rates of recurrent VTE per 100 person-years after the cessation of anticoagulant therapy by duration of therapy.

Duration of anticoagulant therapy (months)	Men with a recurrent VTE (n)	Person-time at risk (months)	Incidence per 100 person-years (95% CI)
≤3	17	88	19.2 (11.2–30.8)
>3 to 6	15	115	13.1 (7.3–21.5)
>6 to 9	20	137	14.7 (8.9–22.6)
>9	18	120	15.0 (8.9–23.7)

*Off-treatment=any time after the end of anticoagulant treatment.

Supplementary Table 7b. Incidence rates of 'on-treatment' and 'off-treatment' major bleeding events per 100 person-years.

	Men with a major bleeding event (n)	Person-time at risk	Incidence per 100 person-years (95% CI)
**During anticoagulant treatment ('on-treatment', n=1413)**			
Intracranial	16	1234	1.30 (0.79–2.12)
Gastrointestinal	33	1225	2.69 (1.92–3.79)
Urogenital	49	1210	4.05 (3.06–5.36)
**Any time after the end of anticoagulant therapy ('off-treatment, n=936)**			
Intracranial	12	1367	0.88 (0.45–1.53)
Gastrointestinal	15	1366	1.10 (0.61–1.81)
Urogenital	25	1361	1.84 (1.19–2.71)

*Off-treatment=any time after the end of anticoagulant treatment.

Supplementary Table 8. Cumulative incidence (%) of recurrent VTE after the cessation of anticoagulant therapy according to the duration of anticoagulant therapy for the index VTE.

Days after end of anticoagulant therapy	Duration of anticoagulant therapy (days)							
	≤3 months N=102		>3 to 6 months N=127		>6 to 9 months N=150		>9 months N=131	
	n	Cumulative incidence (95% CI), %	n	Cumulative incidence (95% CI), %	n	Cumulative incidence (95% CI), %	n	Cumulative incidence (95% CI), %
90	8	7.8 (3.4–14.9)	10	7.9 (3.8–14.0)	6	4.0 (1.5–8.5)	6	4.6 (1.7–9.7)
180	14	13.7 (7.7–22.0)	10	7.9 (3.8–14.0)	13	8.7 (4.7–14.4)	9	6.9 (3.2–12.6)
270	17	16.7 (10.0–25.3)	14	11.0 (6.2–17.8)	18	12.0 (7.3–18.3)	13	9.9 (5.4–16.4)
360	17	16.7 (10.0–25.3)	15	11.8 (6.8–18.7)	20	13.3 (8.3–19.8)	18	13.7 (8.4–20.8)

*510 men with VTE and at least one year of follow-up after the end of therapy.

Supplementary Table 9. Cumulative incidence (%) of on-treatment and off-treatment major bleeding.

	1-year cumulative incidence (95% CI)											
	Intracranial bleeding				Gastrointestinal bleeding				Urogenital bleeding			
	On-treatment n (%)		Off-treatment n (%)		On-treatment n (%)		Off-treatment n (%)		On-treatment n (%)		Off-treatment n (%)	
**Time at risk (days)**												
90	9	0.6 (0.3–1.2)	6	0.6 (0.2–1.4)	15	1.1 (0.6–1.7)	1	0.1 (0.0–0.6)	25	1.8 (1.1–2.6)	7	0.7 (03-1.5)
180	10	0.7 (0.3–1.3)	8	0.9 (0.4–1.7)	16	1.1 (0.6–1.8)	5	0.5 (0.2–1.2)	31	2.2 (1.5–3.1)	8	0.9 (0.4-1.7)
270	12	0.8 (0.4–1.5)	10	1.1 (0.5–2.0)	19	1.3 (0.8–2.1)	7	0.7 (0.3–1.5)	39	2.8 (2.0–3.8)	13	1.4 (0.7-2.4)
360	13	0.9 (0.5–1.6)	11	1.2 (0.6–2.1)	24	1.7 (1.1–2.5)	8	0.9 (0.4–1.7)	43	3.0 (2.2–4.1)	15	1.6 (0.9-2.6)

VTE, venous thromboembolism

Note, follow-up for on-treatment events was from the index VTE until the bleeding event or the end of anticoagulant treatment (n=1413). Follow-up for off-treatment events was from the end of the anticoagulant treatment to the bleeding event or the end of follow-up (n=936)
